# Health impact assessment of air pollution in Shiraz, Iran: a two-part study

**DOI:** 10.1186/2052-336X-11-11

**Published:** 2013-06-28

**Authors:** Ehsan Gharehchahi, Amir Hossein Mahvi, Hassan Amini, Ramin Nabizadeh, Ali Asghar Akhlaghi, Mansour Shamsipour, Masud Yunesian

**Affiliations:** 1 Department of Environmental Health Engineering, School of Public Health, Tehran University of Medical Sciences, Tehran, Iran; 2 Institute for Environmental Research (IER), Tehran University of Medical Sciences, Tehran, Iran; 3 Kurdistan Environmental Health Research Center, Kurdistan University of Medical Sciences, Sanandaj, Iran; 4 Department of Epidemiology and Biostatistics, School of Public Health, Tehran University of Medical Sciences, Tehran, Iran; 5 Department of Epidemiology and Reproductive Health, Reproductive Epidemiology Research Center, Royan Institute, ACECR, Tehran, Iran; 6 Occupational Sleep Research Center, Tehran University of Medical Sciences, Tehran, Iran; 7 Center for Air Pollution Research (CAPR), Institute for Environmental Research (IER), Tehran University of Medical Sciences, Tehran, Iran

**Keywords:** Air Pollution, AirQ, Cardiovascular Diseases, COPD, Excess Hospitalizations, Health Impact Assessment, Iran, Particulate Matter, Respiratory, Shiraz

## Abstract

We aimed to assess health-impacts of short-term exposure to the air pollutants including PM_10_, SO_2_, and NO_2_ in Shiraz, Iran in a two-part study from 2008 to 2010. In part I, local relative risks (RRs) and baseline incidences (BIs) were calculate using generalized additive models. In part II, we estimated the number of excess hospitalizations (NEHs) due to cardiovascular diseases (CDs), respiratory diseases (RDs), respiratory diseases in elderly group (RDsE—people older than 65 years old), and chronic obstructive pulmonary diseases (COPDs) as a result of exposure to air pollutants using AirQ model, which is proposed approach for air pollution health impact assessment by World Health Organization. In part I, exposure to increase in daily mean concentration of PM_10_ was associated with hospitalizations due to RDs with a RR of 1.0049 [95% confidence interval (CI), 1.0004 to 1.0110]. In addition, exposure to increase in daily mean concentration of SO_2_ and NO_2_ were associated with hospitalizations due to RDsE and COPDs with RRs of 1.0540 [95% CI, 1.0050 to 1.1200], 1.0950 [95% CI, 1.0700 to 1.1100], 1.0280 [95% CI, 1.0110 to 1.0450] and 1.0360 [95% CI, 1.0210 to 1.0510] per 10 μg/m^3^ rise of these pollutants, respectively. In part II, the maximum NEHs due to CDs because of exposure to PM_10_ were in 2009—1489 excess cases (ECs). The maximum NEHs due to RDs because of exposure to PM_10_ were in 2009—1163 ECs. Meanwhile, the maximum NEHs due to RDsE and COPDs because of exposure to SO_2_ were in 2008, which are 520 and 900 ECs, respectively. In conclusion, elevated morbidity risks were found from acute exposure to air pollutants.

## Introduction

Cardiovascular and respiratory diseases, such as chronic obstructive pulmonary diseases (COPDs), are one of the leading causes of mortality and disease burden (e.g., disability-adjusted life years (DALYs)), globally [1,2]. They are increasingly worldwide most prevalent health problems and albeit variety of risk factors have been recognized and introduced as the most common causes of commence or exacerbation, the role of air pollution is irrefutable [3-5]. In fact, many time-series and case-crossover studies [6-14] have demonstrated the contribution of air pollutants in hospitalizations, morbidities, and mortalities (i.e., years of life lost—YLL) due to cardiopulmonary diseases, which have been approved by large prospective cohort studies [15,16]. With these as backdrop, the importance of health impact assessment (HIA) of air pollutants in local-scales is obvious to quantify these health impacts, and to develop successful and effective management schemes to reduce YLL and years of life disabled (YLD)—DALYs.

Shiraz—the capital city of Fars province in Southwest of Iran—is one of the largest cities in Iran that is in counter with high amounts of air pollution since last decades as a result of population growth, urbanization, and hence, increased traffic-related air pollution. Besides, the high influxes of air pollutants in last decade through dust storms, well known as Middle Eastern Dust (MED) events, have deteriorated the welkin dramatically. Here, the authors aimed to assess the health impacts of short-term exposure to air pollutants in Shiraz city in a two-part study from 2008 to 2010.

## Materials and methods

This study was conducted in two parts in Shiraz city of Iran—parts I and II. In part I, local relative risks (RRs) and baseline incidences (BIs) were calculate. In part II, calculation and comparison of number of the excess cardiovascular and respiratory hospitalizations because of exposure to air pollutants were done first by using existed default relative risk (RR) and baseline incidence (BI) of the air quality health impact assessment software AirQ 2.2.3, which is proposed approach by World Health Organization (WHO) for HIA [17]; and second by calculated RR and BI of part I or by applying a combination of these RRs and BIs in AirQ model.

### Part I: calculation of local RRs and BIs

In this part, associations of exposure to air pollutants and hospitalizations due to cardiovascular and respiratory causes were examined in Shiraz city as the target population from 2008 to 2010.

### Study area and demographic parameters

**Figure 1:**
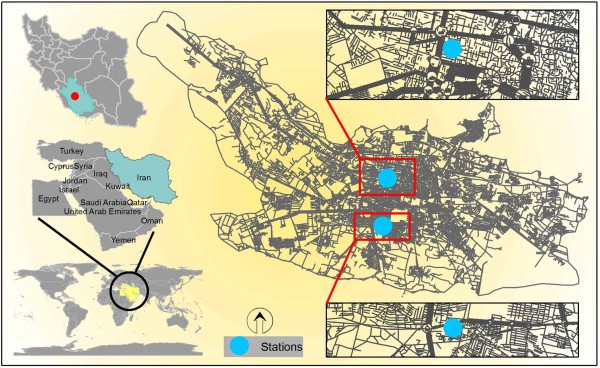
The study area of Shiraz, Iran and location of the air quality monitoring stations.

Shiraz is the capital city of Fars Province in Southwest of Iran (Figure 1). It is located in the latitude of 29°36′ N and the longitude of 52°32′ E with average elevation of 1500 m above sea level. According to the latest census report by Statistical Centre of Iran (SCI) in 2005, the population is 1,2 million people, which this number has been estimated to increase to 1,3 million people in 2010. The population of people older than 65 years old is about 85913. Meanwhile, Shiraz has a population growth rate of 1.3% based on the estimates of SCI [18].

### Air pollution data

At the time of conducting this study there were two fixed air pollution monitors belonged to the Fars's Department of Environment (F-DOE) (Figure 1). Hourly air pollution data including particulate matter with aerodynamic diameter less than 10 μm (PM_10_), sulfur dioxide (SO_2_), and nitrogen dioxide (NO_2_) obtained from F-DOE. Thereafter, 24-hour means were calculated for each pollutant.

### Morbidity cases and classification

The numbers of daily hospitalizations due to cardiovascular and respiratory causes were obtained from Medical Records Departments (MRD) of Shiraz hospitals. The MRDs of Shiraz hospitals provided morbidity cases in line with taxonomy of the International Classification of Diseases (ICD-10). According to the ICD-10, I00 to I99 and J00 to J99 codes have been defined for cardiovascular and respiratory diseases, respectively [19].

### Correlation evaluation and RR calculation

Correlation evaluation and RR calculation were done by applying Poisson regression with logarithmic link in generalized additive models (GAM). The GAM is expressed as the following equation [12]:

(1) $$\log\left[E\left(Y\right)\right]=\beta_{0}+\beta_{1}\times \mathrm{pollutant}+S_{i}\left(X_{i}\right)+\cdot \cdot \cdot +S_{p}\left(X_{p}\right)$$

Where *Y* denotes the number of daily hospitalizations; *E(Y)* denotes the number of expected cases; *X*_*i*_, *i = 1,…,p* which can be temperature, relative humidity, time, etc.; and *S*_*i*_, *i = 1,…,p* denotes smooth functions.

In order to analyze the data, first, quality of them were checked for missing data and errors using SPSS and Microsoft Office Excel, and resolved as possible. In this part of the study, the effects of meteorology variables including temperature and relative humidity; day of the week including working days, holidays, and days after holidays; and time were entered to the model as confounding factors and their effects were removed on number of hospitalizations using smooth function to calculate net effect of pollutant on the number of hospitalizations. In order to adjust the effect of those predictor variables in which affect response variable with lag of several days, smooth function was applied on auto regressive values. Finally, GAM models were fitted on the data using "mgcv" package in R statistical software [20,21].

### Part II: health impact assessment of air pollution

The main aim of this part of the study was HIA of air pollution on the target population using AirQ 2.2.3 software. This software has been developed to estimate the health impacts of exposure to specific air pollutants on a resident population in a certain area and period. In this software, HIA of air pollutants is standing on calculation of attributable proportion (AP) in which AP is fraction of health consequences in a specific population that can be attributed to a specific air pollutant exposure with this notion that there is proven causative correlation between health consequences and air pollutant exposure [22,23]. The AP is calculated as the following equation [23]:

(2) $$\mathrm{AP}=\frac{\Sigma \left\{\left[\mathrm{RR}\left(c\right)-1\right]\times P\left(c\right)\right\}}{\Sigma \left[\mathrm{RR}\left(c\right)\times P\left(c\right)\right]}\;$$

Where RR denotes the relative risk for a given health endpoint, in category “c” of exposure, obtained from the concentration–response functions derived from wide literature (*i.e.,* current existent epidemiological studies) and *P(c)* denotes the proportion of the population in category “c” of exposure.

The rate attributable to the exposure can be calculated as the following equation if the baseline frequency of the health endpoint is known in the population:

(3) IE=I×AP

Where IE denotes the rate of the health outcome attributable to the exposure and *I* denotes the baseline frequency of the health endpoint in the population.

Finally, the number of cases attributable to the exposure can be estimated as the following equation knowing the size of the population:

(4) NE=IE×N

Where NE denotes the number of cases attributed to the exposure and N denotes the size of the population investigated.

In this research, air quality, health outcomes, and exposed population data were entered to the software for the period of 2008 to 2010. Moreover, estimation of the excess hospitalizations due to cardiovascular diseases, respiratory diseases, COPDs, and respiratory diseases in elderly group—people older than 65 years-old—as a result of short-term exposure to PM_10_, SO_2_, and NO_2_ were calculated using RR and BI of WHO, RR and BI of part I, RR of WHO and BI of part I, and RR of part I and BI of WHO for 2008, 2009, and 2010. It is noteworthy that the estimates of AirQ for different air pollutants should not be summed as it leads to overestimation (double counting) of some mortality/morbidity cases [24].

### Input adjustments

HIA by AirQ needs concentration data of pollutants in μg/m^3^ units. Thus, if the measurements of the monitors were reported in parts-per-million (ppm) or parts-per-billion (ppb) units, it would be necessary first to transform ppm or ppb to μg/m^3^ units. In this procedure, there would be some adjustments for pressure and temperature. Hence, the following equation was used to transform ppm to μg/m^3^ units [25]:

(5) $$C\left(\frac{\mu g}{m^{3}}\right)=\frac{C\left(\mathrm{ppm}\right)\times \mathrm{MW}}{V}\times 1000$$

Where *C* denotes the concentration of gaseous composition, *MW* denotes the relative molecular mass of gaseous composition, and *V* denotes the volume of one mole of pure gas at standard temperature and pressure (STP—0°C and 1 atmosphere).

We also made corrections for non-standard temperatures and pressures using Ideal Gas Equation:

(6) $$\frac{P_{1}V_{1}}{T_{1}}=\frac{P_{2}V_{2}}{T_{2}}\;$$

Where *P*_*1*_, *V*_*1*_, and *T*_*1*_ are the initial pressure, volume and absolute temperature and *P*_*2*_, *V*_*2*_, and *T*_*2*_ are the final pressure, volume and absolute temperature.

## Results

### Results of part I

**Figure 2:**
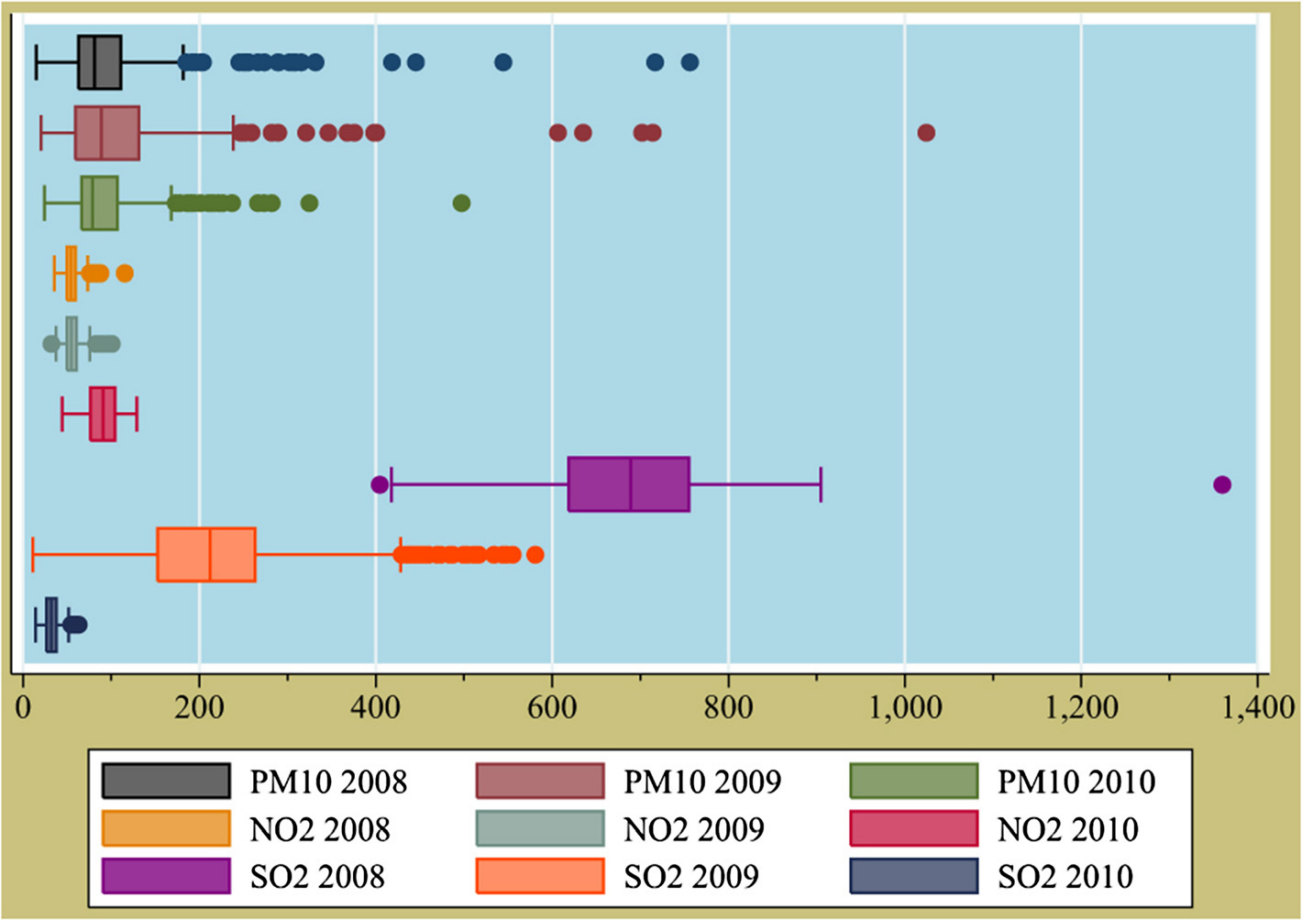
**Annual concentrations of air pollutants (μg/m**^**3**^**) in Shiraz, Iran from 2008 to 2010.**

Figure 2 shows descriptive statistics of air quality in Shiraz in 2008 to 2010. The maximum daily and annual mean PM_10_ concentrations in 2009 were 1024.4 and 111.3 μg/m^3^, respectively. In 2008, the annual mean concentration of SO_2_ was 674.9 μg/m^3^, which is much higher than daily means in 2009 and 2010. In addition, the maximum annual mean concentration of NO_2_ concentration was in 2010, which was 90.0 μg/m^3^.

In Table 1, calculated RR and BI of the part I with 95% confidence interval (CI) in 10 μg increase of daily mean of PM_10_, SO_2_, and NO_2_ pollutants have been tabulated in relation with excess hospitalization cases due to cardiovascular diseases, respiratory diseases, respiratory diseases in elderly group, and COPDs. As can be seen in Table 1, no BI has been reported for respiratory diseases in elderly group so that calculation of excess cases due to short-term exposure to SO_2_ and NO_2_ pollutants have been calculated considering RR of WHO & BI of part I; and RR & BI of part I. According to the results of part I, association of PM_10_ and hospitalizations due to cardiovascular diseases was not significant (*p > 0.05*). However, there was a significant relationship between PM_10_ and hospitalizations due to respiratory diseases (*p = 0.048*). In addition, according to the findings of part I, there was significant association between SO_2_ and NO_2_ pollutants and hospitalizations due to respiratory diseases in elderly group and COPDs (*p < 0.001*).

**Table 1:** **Relative risks with 95% confidence interval for each 10 μg/m**^**3 **^**increase in daily mean concentrations of PM**_**10**_**, SO**_**2**_**, and NO**_**2**_

**Health end point**	**PM**_**10**_		**SO**_**2**_		**NO**_**2**_		**BI per 100000**	
	**WHO RR**	**Part I RR**	**WHO RR**	**Part I RR**	**WHO RR**	**Part I RR**	**WHO**	**Part I**
	**(95% CI)**	**(95% CI)**	**(95% CI)**	**(95% CI)**	**(95% CI)**	**(95% CI)**		
Hospital admissions due to CDs	1.009	1.002	-*	-	-	-	436	1447
	(1.006 to 1.013)	(1.0001 to 1.0040)						
Hospital admissions due to RDs	1.0080	1.0049	-	-	-	-	1260	849
	(1.0048 to 1.0112)	(1.0004 to 1.0110)						
Hospital admissions due to RDsE**	-	-	1.004	1.054	1.0038	1.028	-***	61
			(1.001 to 1.009)	(1.005 to 1.120)	(1.0000 to 1.0120)	(1.011 to 1.045)		
Hospital admissions due to COPDs	-****	-	1.0044	1.095	1.0038	1.036	101.4	92
			(1.0000 to 1.0110)	(1.070 to 1.110)	(1.0004 to 1.0094)	(1.021 to 1.051)		

Abbreviations: *BI* baseline incidence, *CDs* cardiovascular diseases, *CI* confidence interval, *COPDs* chronic obstructive pulmonary diseases, *NO*_*2*_ nitrogen dioxide, *PM*_*10*_ particulate matter with aerodynamic diameter less than 10 μm, *RDs* respiratory diseases, *RDsE* respiratory diseases in elderly group (people older than 65 years-old), *RR* relative risk, *SO*_*2*_ sulfur dioxide, *WHO* World Health Organization.

*There are no RRs for cardiovascular and respiratory diseases due to short-term exposure to SO_2_ and NO_2_ in AirQ software so that they were not calculated in part I of the study.

** Based on classification of AirQ.

***The BI has not been presented in AirQ for respiratory diseases in elderly group.

**** There is no RR for COPDs due to short-term exposure to PM_10_ in AirQ so that it is not calculated in part I of the study.

### Results of part II

In Table 2, the numbers of excess hospitalization cases due to cardiovascular and respiratory diseases as a result of short-term exposure to PM_10_ in 2008 to 2010 have been tabulated. As can be seen, the maximum number of excess hospitalizations due to cardiovascular and respiratory diseases is related to RR of WHO & BI of part I; and RR & BI of WHO, respectively. According to the results of this table, the maximum number of excess hospitalizations due to cardiovascular and respiratory diseases is because of short-term exposure to PM_10_ in 2009.

**Table 2:** **Number of excess hospitalizations due to cardiovascular and respiratory diseases as a result of short-term exposure to PM**_**10 **_**in 2008 to 2010**

**Year**	**Health end point**	**Number of excess cases**			
		**WHO RR & BI**	**WHO RR & Part I BI**	**Part I RR & BI**	**Part I RR & WHO BI**
2008	Hospital admissions due to cardiovascular diseases	419	1389	328	99
	Hospital admissions due to respiratory diseases	1084	731	460	682
2009	Hospital admissions due to cardiovascular diseases	449	1489	353	106
	Hospital admissions due to respiratory diseases	1163	783	494	733
2010	Hospital admissions due to cardiovascular diseases	388	1287	302	91
	Hospital admissions due to respiratory diseases	1004	677	424	630

Abbreviations: *WHO* World Health Organization, *RR* relative risk, *BI* baseline incidence.

In Table 3, the numbers of excess hospitalizations due to respiratory diseases in elderly group and COPDs because of short-term exposure to SO_2_ have been calculated based on a combination of RR & BI of WHO (default values). According to the results of this table, the maximum numbers of hospitalizations due to respiratory diseases are in elderly group and hospitalizations due to COPDs because of short-term exposure to SO_2_ in 2008.

**Table 3:** **Number of excess hospitalizations due to respiratory diseases as a result of short-term exposure to SO**_**2 **_**in 2008 to 2010**

**Year**	**Health end point**	**Number of excess cases**			
		**WHO RR & BI**	**WHO RR & Part I BI**	**Part I RR & BI**	**Part I RR & WHO BI**
2008	Hospital admissions due to RDsE	-	110	520	-
	Hospital admissions due to COPDs	198	180	900	992
2009	Hospital admissions due to RDsE	-	60	410	-
	Hospital admissions due to COPDs	108	98	776	855
2010	Hospital admissions due to RDsE	-	7	137	-
	Hospital admissions due to COPDs	13	12	208	229

Abbreviations: *WHO* World Health Organization, *RR* relative risk, *BI* baseline incidence, *RDsE* respiratory diseases in elderly group (people older than 65 years-old), *COPDs* chronic obstructive pulmonary diseases.

In Table 4, the number of excess hospitalizations due to respiratory diseases in elderly group and COPDs as a result of short-term exposure to NO_2_ have been calculated based on a combination of RR & BI of WHO (default values). According to the results of this table, the maximum numbers of hospitalizations due to respiratory diseases are in elderly group and hospitalizations due to COPDs because of short-term exposure to NO_2_ in 2010.

**Table 4:** **Number of excess hospitalizations due to respiratory diseases as a result of short-term exposure to NO**_**2 **_**in 2008 to 2010**

**Year**	**Health end point**	**Number of excess cases**			
		**WHO RR & BI**	**WHO RR & Part I BI**	**Part I RR & BI**	**Part I RR & WHO BI**
2008	Hospital admissions due to RDsE	-	13	57	-
	Hospital admissions due to COPDs	21	19	160	177
2009	Hospital admissions due to RDsE	-	13	89	-
	Hospital admissions due to COPDs	22	20	166	183
2010	Hospital admissions due to RDsE	-	23	145	-
	Hospital admissions due to COPDs	39	35	265	292

Abbreviations: *WHO* World Health Organization, *RR* relative risk, *BI* baseline incidence, *RDsE* respiratory diseases in elderly group (people older than 65 years-old), *COPDs* chronic obstructive pulmonary diseases.

## Discussion

In this research, the authors tried to quantify effects of short-term exposure to specific air pollutants—PM_10_, SO_2_, and NO_2_—on some health consequences including cardiovascular and respiratory diseases, such as COPDs, using AirQ model in different scenarios. In other words, we tried to estimate excess hospitalization cases due to cardiovascular diseases, respiratory diseases, respiratory diseases in elderly group, and COPDs due to short-term exposure to air pollutants. Noteworthy, estimation of number of excess hospitalizations due to respiratory diseases as a result of short-term exposure to SO_2_ and NO_2_ are categorized in three age-groups including < 15, 15–65, and ≥ 65 years-old. Notwithstanding, in this study—part I—categorization of patients was in two groups (< 65 and ≥ 65 years-old). Thus, calculation of RR and BI for ≤ 15 and 15–65 years-old age-groups was impossible so that calculation of RR, BI, and number of excess hospitalizations due to respiratory diseases was done for ≥ 65 years-old age-group.

As illustrated in Figure 2, the annual mean concentration of PM_10_ in 2008 to 2010 has been 102.5, 111.3, and 92.1 μg/m^3^, respectively. Likewise, analyses of PM_10_ concentrations in 2008 to 2010 revealed occurrence of MED events, especially in 2009, which can affect results of Part I. With this in mind, indeed, AirQ software has been developed based on RRs of exposure to traffic-related air pollutants so that RRs of this software may not necessarily be applicable for HIA of MED events [24]. Meanwhile, the annual mean concentration of SO_2_ in 2008 to 2010 has been 674.8, 209.4, and 32.7 μg/m^3^, respectively, which shows a decreasing slope. This may be due to the decrease of sulfur in the fuel and/or increase of vehicles that use compressed natural gas (CNG) for gas up—albeit this point has been confirmed by F-DOE that those vehicles that use gasoline for gas up are responsible for SO_2_ pollution in Shiraz. In addition, the annual mean concentration of NO_2_ during the mentioned period has been 55.0, 55.8, and 90.0 μg/m^3^, respectively, which evidently is increased and supports the notion of increase in use of CNG instead of gasoline since combustion of CNG produce more nitrogen oxides.

As shown in Table 1, the calculated BI per 100,000 people in part I of the study due to respiratory diseases, is 25% less than BI of WHO whilst this measure for cardiovascular diseases is 4.9 fold of WHO's BI. While these may be true, it is obvious that Shiraz is medical hub in Southwest of Iran and it hosts many patients from outside of the city. Since there were no adequate and accurate addresses for patients by MRDs of hospitals, all admissions were entered to the calculation of BI, which this may be the reason for obtaining greater BI for part I rather than BI of WHO.

There is no BI value for hospitalizations due to respiratory diseases in ≥ 65 years-old age-group as a result of short-term exposure to SO_2_ and NO_2_ in the AirQ software so that the number of excess hospitalization cases were calculated by a combination of WHO's RR, part I's RR, and BI of part I of the study.

HIA of air pollution in Shiraz city using AirQ software revealed that the share of admissions for cardiovascular diseases as a result of exposure to PM_10_ during 2008 to 2010 and considering to RR & BI of WHO are 2.3%, 2.1%, and 1.5% out of all shares of admissions due to cardiovascular diseases, respectively. Meanwhile, the share of admissions for cardiovascular diseases as a result of exposure to PM_10_ during 2008 to 2010 and considering to RR & BI of part I are 1.8%, 1.6%, and 1.2% out of all shares of admissions due to cardiovascular diseases, respectively, which are lower than estimates by using WHO's RR & BI. Furthermore, results revealed that the share of admissions for respiratory diseases as a result of exposure to PM_10_ during 2008 to 2010 and considering to RR & BI of WHO are 19.0%, 15.6%, and 10.8% out of all shares of admissions due to respiratory diseases, respectively. Also, the shares of admissions for respiratory diseases as a result of exposure to PM_10_ during 2008 to 2010 and considering to RR & BI of part I are 8.1%, 6.6%, and 4.6% out of all shares of admissions due to respiratory diseases, respectively, and are lower than estimates by using WHO's RR and BI.

As tabulated in Table 2, in line with estimates, the maximum number of excess hospitalizations due to cardiovascular and respiratory diseases attributable to PM_10_ exposure have been occurred in 2009, which is due to high concentrations of PM_10_ during most times of the year. Indeed, it is important to note that albeit calculated RRs for increase in 10 μg/m^3^ of PM_10_ are small for individuals, there would be extensive impacts in general population when they have exposure with PM_10_.

The shares of admissions due to respiratory diseases in elderly group as a result of exposure to SO_2_ during 2008 to 2010 and considering to RR of WHO & BI of part I are 6.4%, 2.7%, and 0.3% out of all shares of admissions due to respiratory diseases in elderly group, respectively. Also, the shares of admissions due to respiratory diseases in elderly group as a result of exposure to SO_2_ during 2008 to 2010 and considering to RR & BI of part I are 30.3%, 18.3%, and 4.9% out of all shares of admissions due to respiratory diseases in elderly group, respectively.

The shares of admissions for COPDs as a result of exposure to SO_2_ during 2008 to 2010 and considering to RR and BI of WHO are 12.4%, 5.3%, and 0.5% out of all shares of admissions due to COPDs, respectively. Also, the share of admissions for COPDs as a result of exposure to SO_2_ during 2008 to 2010 and considering to RR & BI of part I are 56.2%, 37.8%, and 8.3% out of all shares of admissions due to COPDs, respectively. In fact, various factors, such as smoking, occupational exposures, inappropriate diet, indoor air pollution, exposure to NO_2_ and particulate matters can influence incidence of COPDs and RDs, especially in elderly people, but exposure to air pollutants can exacerbate these diseases as predisposing factors [26-34].

The share of admissions due to respiratory diseases in elderly group as a result of exposure to NO_2_ during 2008 to 2010 and considering to RR of WHO & BI of part I are 0.8%, 0.6%, and 0.8% out of all shares of admissions due to respiratory diseases in elderly group, respectively. Also, the shares of admissions due to respiratory diseases in elderly group as a result of exposure to NO_2_ during 2008 to 2010 and considering to RR & BI of part I are 3.3%, 4.0%, and 5.2% out of all shares of admissions due to respiratory diseases in elderly group, respectively.

The shares of admissions due to COPDs as a result of exposure to NO_2_ during 2008 to 2010 and considering to RR of WHO and BI of part I are 1.3%, 1.1%, and 1.6% out of all shares of admissions due to COPDs, respectively. Also, the shares of admissions due to COPDs as a result of exposure to NO_2_ during 2008 to 2010 and considering to RR & BI of part I are 10.0%, 8.1%, and 10.6% out of all shares of admissions due to COPDs, respectively.

As tabulated in Tables 1, 2, 3, and 4, combination of RRs and BIs of WHO and part I give different results. There is no doubt, by all means, that the number of hospitalizations would decrease by abatement of air pollutants concentrations, even in using combinations of all RRs and BIs.

Many researchers, worldwide, have used the AirQ model. The majority of them, however, have focused on short-term effects of air pollutants on mortality due to cardiovascular and respiratory diseases [5,23,35-37]. For instance, Naddafi et al. (2012) have estimated that short-term exposure to air pollutants including PM_10_, SO_2_, NO_2_, and O_3_ in Tehran caused 2194, 1458, 1050, and 819 excess total mortality cases, respectively [5].

## Conclusions

This study was the first attempt to assess health impacts of air pollution in Shiraz, Iran. Although the results of this study are in line with results of other researches around the world, since the geographic, demographic, and climate characteristics are different, there is still high need to further studies to specify local RRs and BIs. As mentioned above, air quality affects daily hospital admissions dramatically. Accordingly, cost-effective measures and management schemes should be considered to abate air pollution concentrations and/or reduce exposure of general population to air pollutants.

## Abbreviations

AP: Attributable proportion; BI: Baseline incidence; COPDs: Chronic obstructive pulmonary diseases; CI: Confidence interval; GAMs: Generalized additive models; HIA: Health impact assessment; ICD: International classification of diseases; NO2: Nitrogen dioxide; PM10: Particulate matter with aerodynamic diameter less than 10 μm; RR: Relative risk; SO2: Sulfur dioxide; WHO: World Health Organization

## Competing interests

The authors declare they have no potential or actual competing financial or personal interests.

## Authors’ contributions

MY, AHM, and RN participated in the design of the study and supervised the work. EG, HA, AAA, MS, and MY participated in the design of the study, and/or statistical analyses, and/or interpreted the analyzed results. HA and EG wrote the first draft. AAA, MS, and MY revised the paper critically for important intellectual content. All authors have read and approved the final manuscript.
